# A modified Larson’s method of posterolateral corner reconstruction of the knee reproducing the physiological tensioning pattern of the lateral collateral and popliteofibular ligaments

**DOI:** 10.1186/1758-2555-4-21

**Published:** 2012-06-13

**Authors:** Yasuo Niki, Hideo Matsumoto, Toshiro Otani, Hiroyuki Enomoto, Yoshiaki Toyama, Yasunori Suda

**Affiliations:** 1 Department of Orthopaedic Surgery, Keio University, 35 Shinanomachi, Shinjuku-ku, Tokyo, 160-8582, Japan

**Keywords:** Posterolateral corner, Lateral collateral ligament, Popliteofibular ligament, Reconstructive technique

## Abstract

**Background:**

Consensus has been lacking as to how to reconstruct the posterolateral corner (PLC) of the knee in patients with posterolateral instability. We describe a new reconstructive technique for PLC based on Larson's method, which reflects the physiological load-sharing pattern of the lateral collateral ligament (LCL) and popliteofibular ligament (PFL).

**Findings:**

Semitendinosus graft is harvested, and one limb of the graft comprises PFL and the other comprises LCL. Femoral bone tunnels for the LCL and popliteus tendon are made at their anatomical insertions. Fibular bone tunnel is prepared from the anatomical insertion of the LCL to the proximal posteromedial portion of the fibular head, which corresponds to the insertion of the PFL. The graft end for popliteus tendon is delivered into the femoral bone tunnel and secured on the medial femoral condyle. The other end for LCL is passed through the fibular tunnel from posterior to anterior. While the knee is held in 90 of flexion, the graft is secured in the fibular tunnel using a 5 mm interference screw. Then, the LCL end is passed into the femoral bone tunnel and secured at the knee in extension.

**Conclusions:**

Differential tension patterns between LCL and PFL is critical when securing these graft limbs. Intrafibular fixation of the graft using a small interference screw allows us to secure these two graft limbs independently with intended tension at the intended flexion angle of the knee.

## Introduction

Generally, posterolateral corner (PLC) reconstruction is performed to treat chronic posterolateral instability in patients with PLC injury. However, consensus has been lacking as to how to reconstruct the PLC. Abundant surgical procedures for PLC have been accumulated, and can be broadly divided into two types: anatomical and non-anatomical. Non-anatomical reconstructions include biceps tenodesis [1,2], arcuate complex [3], proximal bone block advancements [4], and extracapsular iliotibial band sling [5]. However, current techniques have shifted to more anatomical reconstruction of the three major functional components of the PLC: the lateral collateral ligament (LCL), popliteofibular ligament (PFL), and popliteus tendon [6-9]. We have developed a new reconstructive technique for PLC based on Larson’s method [10], which reflects the physiological load-sharing pattern of the LCL and PFL. This technique is less invasive and less technically demanding than current anatomical reconstructive techniques.

## Surgical techniques

The patient is positioned supine on the operating table with an arthroscopic leg holder, after precise diagnosis of posterolateral instability and concomitant injuries. Arthroscopy is performed to identify lateral drive-through sign as well as concomitant disruption of the anterior cruciate ligament (ACL) or posterior cruciate ligament (PCL). If the decision has been made to reconstruct either of the cruciate ligaments, this should be performed first. After cruciate ligament reconstruction, PLC reconstruction is initiated. Semitendinosus (ST) tendon is normally harvested ipsilaterally using a smooth tendon stripper, but if ipsilateral ST tendon is planned for use as either PCL or ACL graft, the graft for PLC is harvested from the contralateral ST tendon. The appropriate length of ST graft for PLC is 16–19 cm, which typically reflects the distance between the femoral and fibular insertions of the PLC plus an additional 30 mm, as both ends of the graft require at least 15 mm each, corresponding to the insertion into bone tunnels. One limb of the graft comprises PFL and the other comprises LCL. A baseball glove suture using FiberWire^TM^ (Arthrex, Naples, FL) is carried used at the both ends of the graft, and one end of the popliteus tendon is connected to an Endobutton^TM^ (Smith & Nephew, Memphis, TN), then placed within antibiotic-soaked gauzes and set aside for later use.

**Figure 1:**
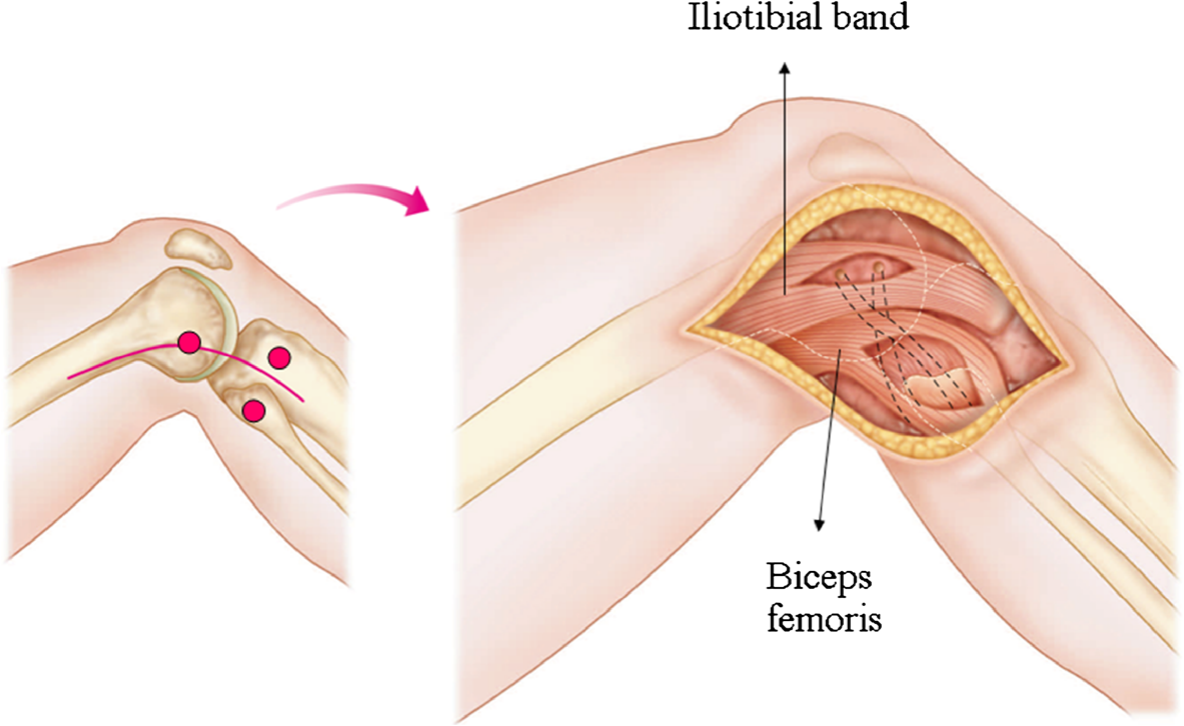
**Schematic representation of surgical landmarks over the skin (left panel).** Incision is made down to the layer of the iliotibial band and biceps femoris to expose the lateral epicondyle and fibular head, respectively (right panel).

**Figure 2:**
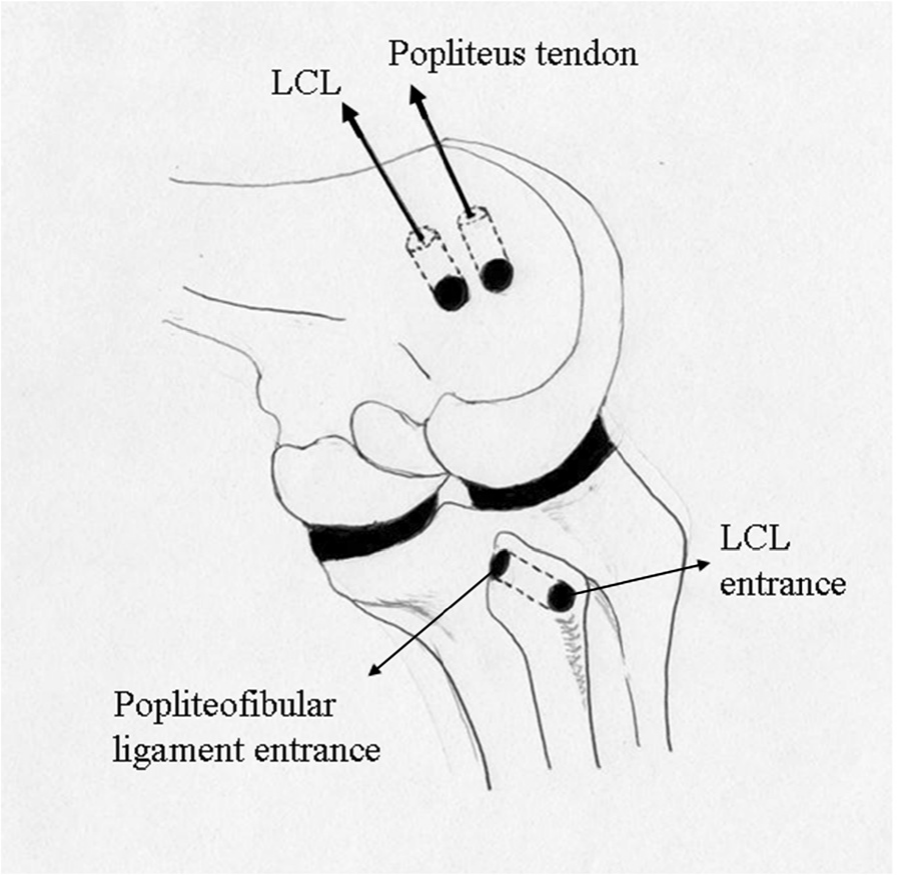
**Preparation of two femoral tunnels and one transfibular tunnel.** Both entrances of the transfibular tunnel ideally correspond to anatomical attachments for the LCL and PFL.

A lateral incision is started just proximal to the lateral femoral epicondyle and followed distally to the midpoint between Gerdy’s tubercle and the fibular head (Figure 1). Further incision is performed down to the deep layer in line with fibers of the iliotibial band from the lateral femoral epicondyle to Gerdy’s tubercle. After femoral attachments of the LCL and popliteus tendon are exposed, both tendon and ligament are taken off the femur. For exposure of fibular attachments of the LCL and PFL, an incision is made on the biceps femoris muscle in line with this fiber so that the fibular head is well exposed. Anatomical insertions of the LCL and popliteus tendon at the lateral femoral condyle are drilled with 2.4-mm guide pins aimed toward the flare of the medial femoral epicondyle, followed by overdrilling using a reamer matched to the diameter of the graft (typically 4–5 mm) (Figure 2).

**Figure 3:**
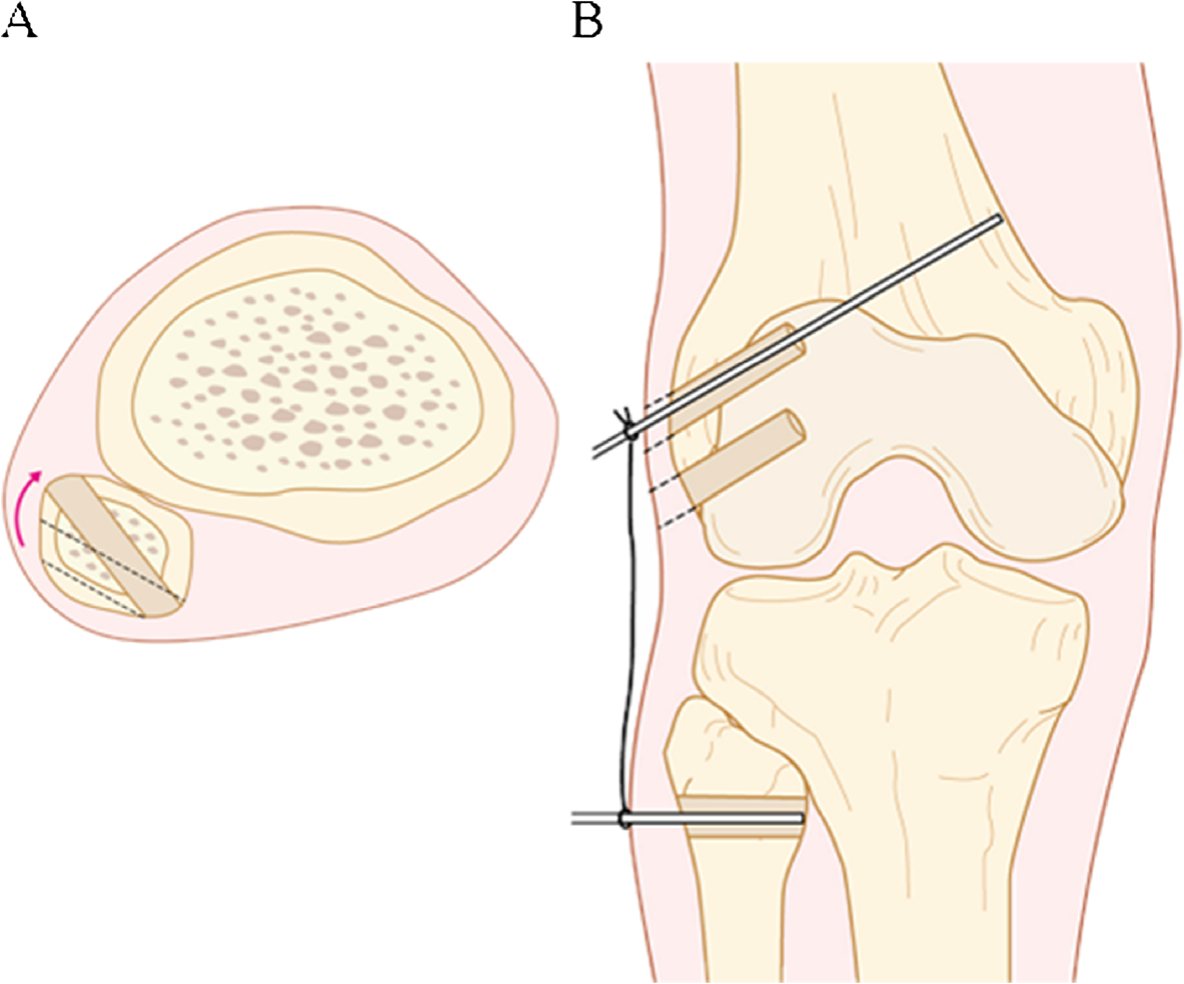
**When the fibular head is small, position of the LCL insertion should preferably be shifted anteriorly to avoid the risk of avulsion of the fibular head by the reamer (A).** Dynamic excursion between the two pins sticking in femoral and fibular attachments should be checked during knee flexion and extension before making the bone tunnels (**B**).

The posterolateral aspect of the fibular head is exposed, and attachments of the LCL and PFL are identified. The peroneal nerve should be identified, marked with tape, and retracted with the biceps muscle. To prepare the fibular tunnel, the starting point is set at the distal anterolateral portion of the fibular head, corresponding to the anatomical insertion of the LCL, and the guide pin should exit the proximal posteromedial portion of the fibular head, which corresponds to the insertion of the PFL. When the patient is of short stature and the fibular head is small, the location of the transfibular tunnel, particularly the LCL insertion site, should be changed from the anatomical site slightly anteriorly to avoid any risk of avulsing the fibular head with the reamer (Figure 3A). At this time, the pattern of length change between the femoral and fibular bone tunnels during knee flexion and extension should be confirmed for both LCL and PFL (Figure 3B). Ideally, the length of LCL will shorten with increasing flexion angle of the knee, while the length of popliteus tendon will increase with increasing flexion angle.

**Figure 4:**
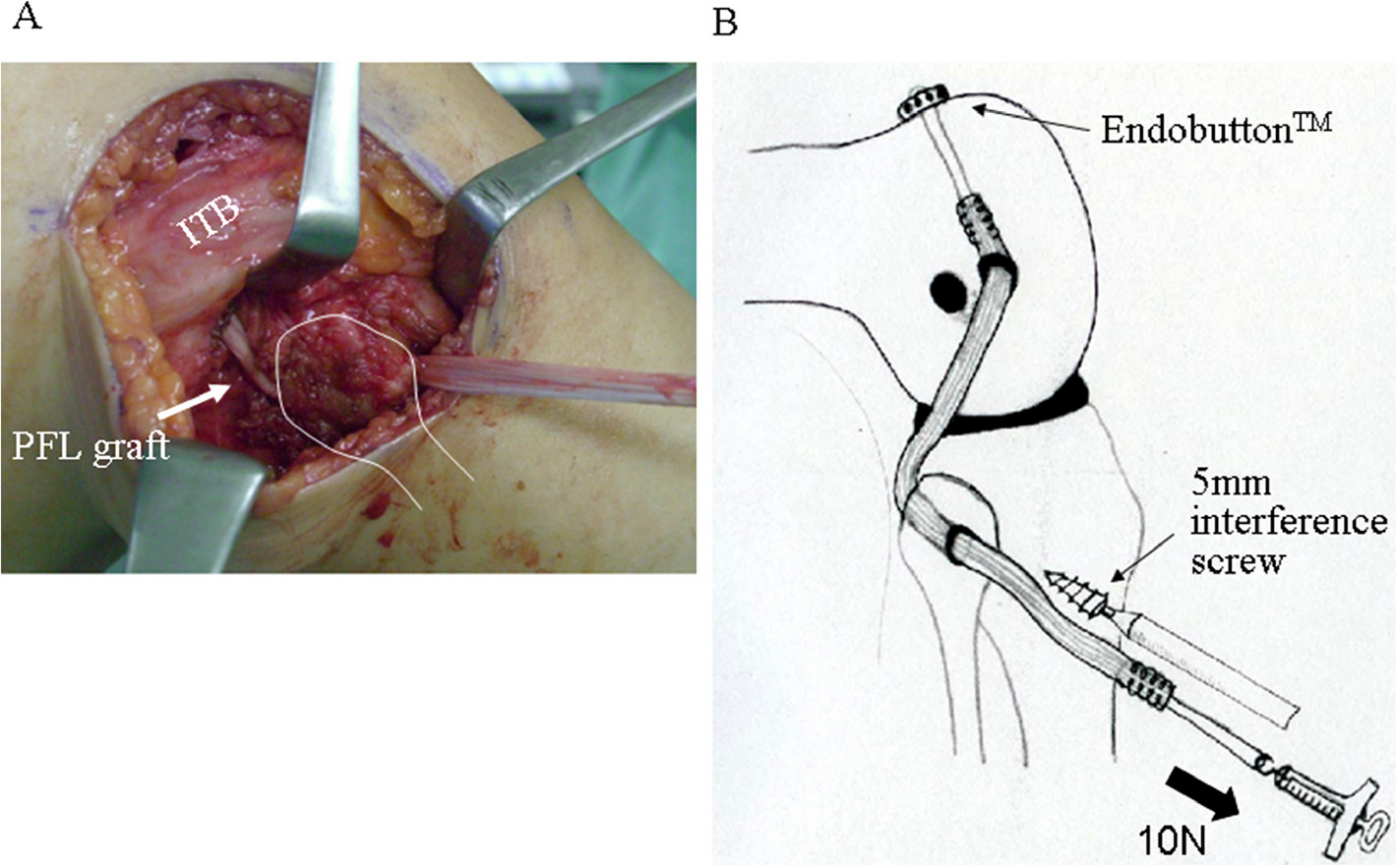
**Semitendinosus tendon graft has been secured within the popliteus femoral tunnel using an Endobutton**^**TM**^**, delivered below the ITB, and passed through the transfibular tunnel (A).** The graft is fixed in the fibular tunnel with a metal interference screw under 10N force of pretension at 90° knee flexion (**B**).

**Figure 5:**
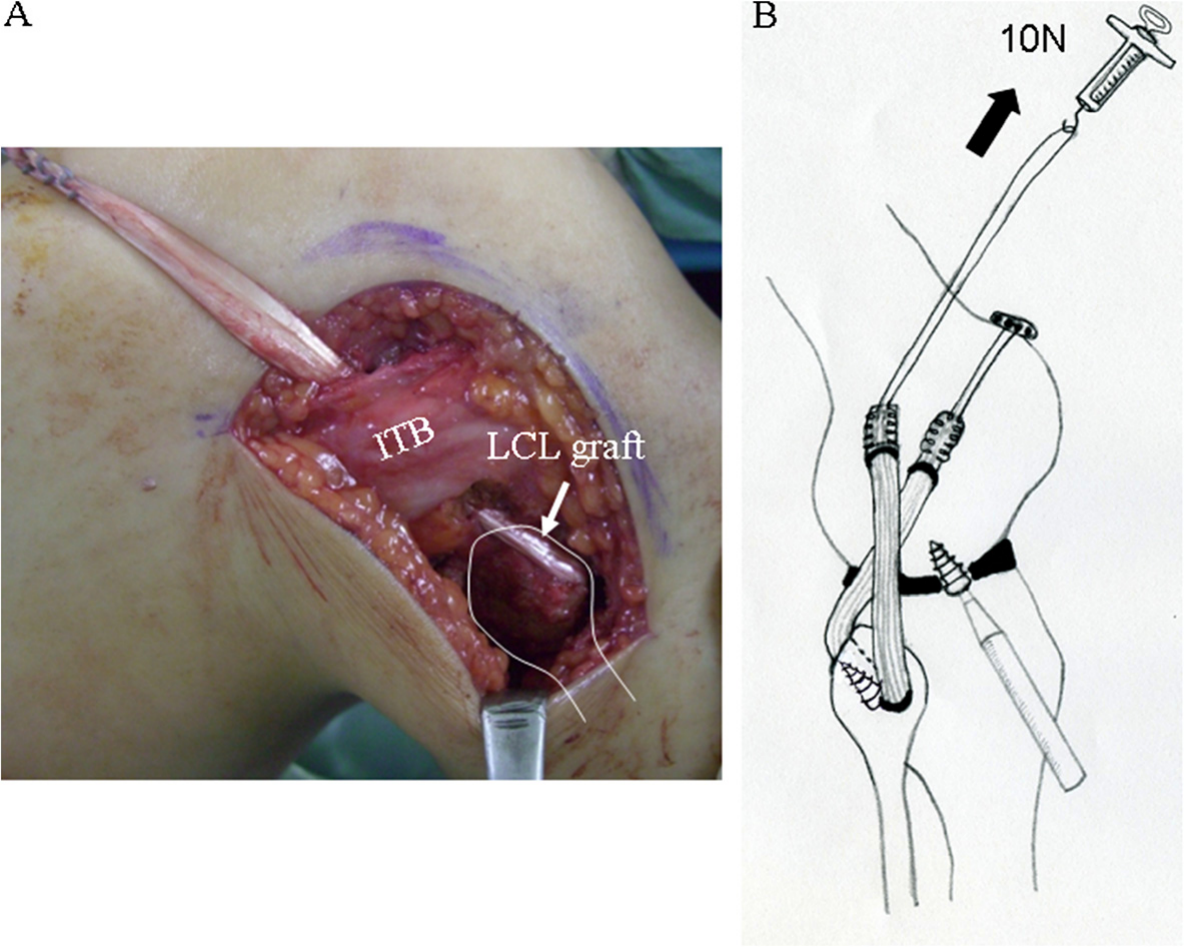
**The graft end for the LCL is delivered under the biceps and ITB (A), and is passed into the femoral bone tunnel from the lateral epicondyle to medial cortex of the femur.** The graft is then secured using an interference screw under 10N force pretension with the knee in extension (**B**).

**Figure 6:**
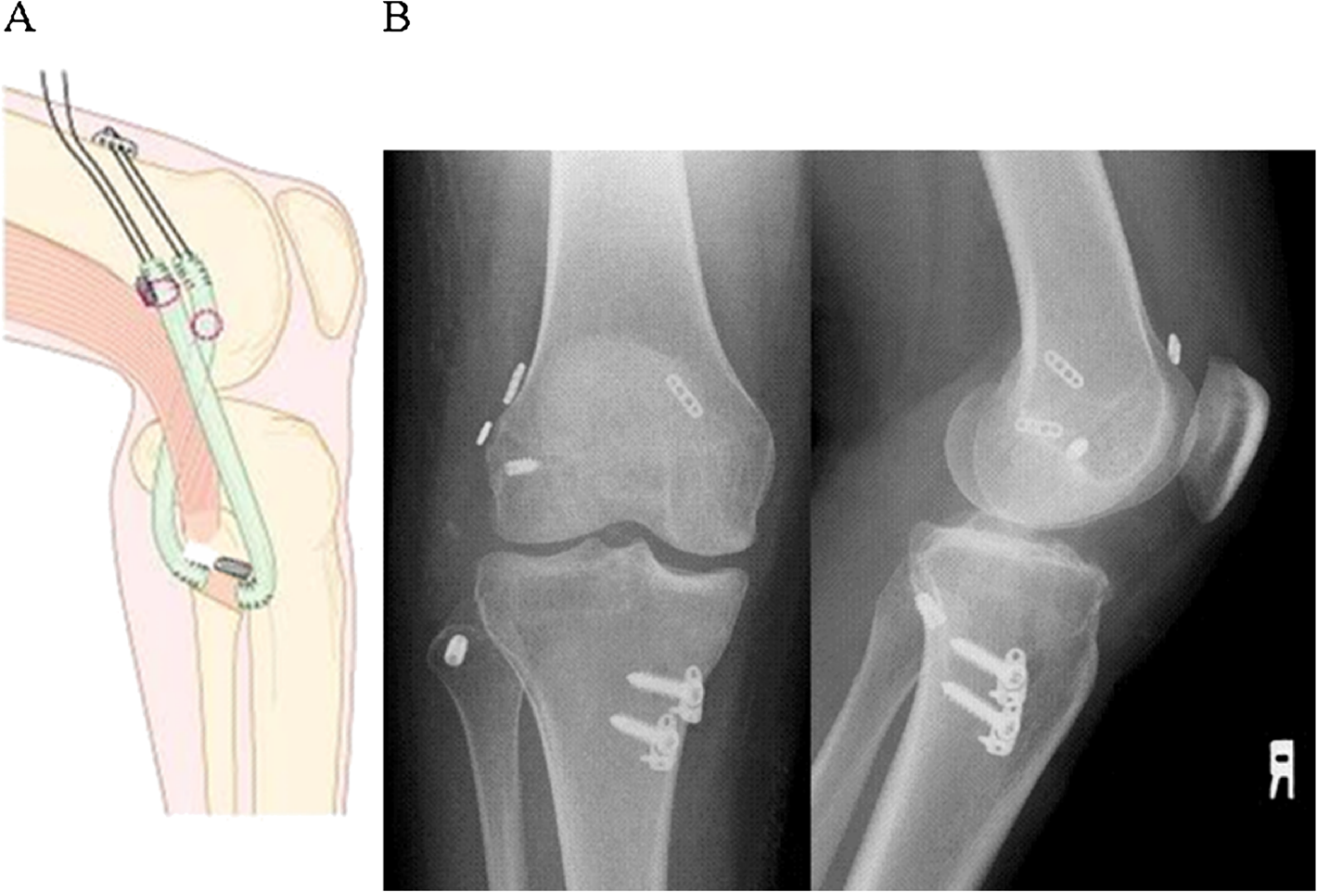
**The reconstructed PFL and LCL cross over each other (A).** Postoperative radiography shows hardware to be used for securing ACL, LCL, and PFL (**B**). When either ACL or PCL is reconstructed simultaneously, particularly with a double-bundle technique, great care should be taken with positioning of each bone tunnel to avoid overlap of these tunnels.

The graft end for PFL connected to the Endobutton^TM^ is delivered into the femoral bone tunnel and flipped on the cortex of the medial femoral condyle. The other end for LCL is then delivered under the iliotibial band and biceps femoris and is passed through the fibular tunnel from posterior to anterior (Figure 4A). As the LCL graft end is strained manually, the knee is taken through several cycles of full flexion and extension. While the knee is held in 90° of flexion and the tibia is in a neutral rotation, the graft is then secured in the fibular tunnel with a metal interference screw 5 mm in diameter (TJ screw; Meira, Nagoya, Japan) with 10 N of force applied on the graft by use of a ligament tensioner (Smith & Nephew Endoscopy) (Figure 4B). The interference screw is inserted into the fibular bone tunnel through anteroposterior direction. The graft end for the LCL is then delivered under the biceps and ITB, and is passed into the femoral bone tunnel from the lateral epicondyle to the medial cortex of the femur (Figure 5)A. The leading FiberWire^TM^ sticks out of the medial skin and is tensioned manually. After performing several cycles of full flexion and extension to provide pretension, graft fixation to the bone is accomplished using the 5-mm interference screw (TJ screw) with 10 N force applied to the graft at the knee in extension and neutral rotation by use of a ligament tensioner (Smith & Nephew Endoscopy) (Figure 5B). To allow adequate interference screw fixation within tunnels, at least 15 mm of graft should be positioned within the tunnel. Full flexion and extension are then verified, and improved knee stability is confirmed, particularly for external rotation at knee in 30° and 90° of flexion and varus stability at the knee in 0° and 30° of flexion. Typically, postoperative radiography shows the Endobutton^TM^ connected to the graft end of the PFL located in the anterolateral cortex of the femur (Figure 6B). Importantly, in cases following either ACL or PCL reconstruction, particularly double-bundle reconstruction, the bone tunnel for the LCL and PFL should be absolutely prevented from overlapping with either of the two bone tunnels for cruciate ligament.

## Postoperative program

The postoperative program is normally dictated by the cruciate ligament reconstruction, particularly for PCL reconstruction. The affected knee joint is immobilized in a hinged knee brace locked in extension for 2 weeks postoperatively. Range of motion (ROM) exercises are initiated using a continuous passive motion (CPM) device and are permitted from 0° to 90° of flexion during weeks 3 and 4. From week 4 on, >90 ° of flexion is permitted. The knee is maintained at 0° except during ROM exercises. A hinged functional brace is used for 3 months postoperatively. Partial weight-bearing with the brace locked in extension is initiated at 2 weeks postoperatively, with gradual progression to full weight-bearing by 4 weeks postoperatively.

Written informed consent was obtained from the patient for publication of this report and any accompanying images.

## Discussion

Injuries to the PLC of the knee can result in severe disability due to both instability and articular cartilage degeneration. These injuries do not commonly occur in isolation, but are usually found in the setting of other injuries, such as ACL or PCL ruptures. Most authorities recommend surgical reconstruction of the PLC in combination with ACL or PCL reconstruction [11-13], since solitary reconstruction of these cruciate ligaments may results in high in situ force in the graft and concomitant PLC reconstruction potentially exerts protective effects on early failure of the cruciate ligament reconstruction.

Historically, numerous techniques for PLC reconstruction have been described, but which technique represents the best method for reconstructing physiologically functional PLC remains controversial. According to the distal insertion site of grafts for PLC, two surgical techniques are available: fibular-based techniques and combined tibial-fibular-based techniques. Larson’s procedure was one of the first fibular-based techniques, and reconstructs the LCL and PFL with distal insertion sites located at the fibula [10]. Larson’s procedure is still widely accepted due to the virtues of being less technically demanding and offering promising clinical results. Our technique was developed based on Larson’s methods, and has been modified to reproduce a physiological tension pattern for LCL and PFL using a single ST autograft.

Tibial-fibular-based techniques have gained increasing attention due to their nature of more anatomical reconstruction capable of reconstructing all three major PLC components at each precise insertion site, but certain investigations have reported that these techniques potentiate overconstraint of posterolateral instability [14,15]. We believe that force distribution between the popliteus complex (PFL and popliteus tendon) and LCL is critical and should be taken into careful consideration when securing these grafts intraoperatively. A previous biomechanical study has reported that the magnitude and distribution of in situ force between the LCL and popliteus complex are affected by knee flexion angle and magnitude of posterior tibial load [16]. LCL represents a larger in situ force near full extension, decreasing with increasing flexion angle of the knee, which may explain the clinical observation that LCL is taut near full extension and relatively lax with the knee in flexion. In contrast, the popliteus complex represents a larger in situ force with the knee in flexion than with the knee in extension [12]. This force distribution pattern was employed in our modified Larson’s procedure, which may thus mimic the physiological load-sharing pattern between LCL and the popliteus complex and avoid overconstraint of external and varus rotations of the tibia. Actually, LCL is secured at full extension with 10 N, whereas the PFL is secured at 90° of knee flexion with 10 N in our procedure. Particular emphasis in our technique is placed on intrafibular fixation of the ST graft using a small interference screw, which allows us to secure two graft limbs for LCL and PFL independently with intended tension at the intended flexion angle of the knee, achieving differential tension patterns for LCL and PFL.

Although favorable short-term results of tibial-fibular-based techniques have been reported [7,8], further studies documenting long-term clinical results are warranted to determine whether tibial-fibular-based techniques represent a standard optimal procedure for PLC reconstruction. At present, controversy remains as to whether all three components of the PLC should be reconstructed. Recent studies have postulated several drawbacks for tibial-fibular-based techniques, including increased technical difficulty and potential overconstraint of external and varus rotations of the knee [14,15]. Veltri and Warren have advocated reconstruction of PFL and LCL as sufficient to adequately control posterolateral instability such as posterior tibial translation and external and varus rotations [17,18], which may support our modified Larson’s method. Moreover, as the popliteus constitutively possesses a muscle belly and acts as a dynamic ligament, it is disputable that popliteus is reconstructed as a static ligament using ST tendon. Our modified Larson’s method has advantages of technical simplicity and reproduction of a more physiological load-sharing pattern among grafts as compared with previously described reconstructive procedures and can offer an acceptable choice to treat chronic posterolateral instability. Further follow-up is needed to ensure that our reconstruction techniques of LCL and PFL are suitable to restore posterolateral instability of the knee.

## Competing interests

The authors declare that they have no competing interests.

## Authors’ contributions

YN and HM have conceived and established the surgical techniques. YN and HE have performed the surgery. YN, HM, and TO have contributed to drafting of the manuscript. YS and YT have contributed to proof check of English. All authors have read and approved the final manuscript.
